# Reduction of Pavlovian Bias in Schizophrenia: Enhanced Effects in Clozapine-Administered Patients

**DOI:** 10.1371/journal.pone.0152781

**Published:** 2016-04-04

**Authors:** Matthew A. Albrecht, James A. Waltz, James F. Cavanagh, Michael J. Frank, James M. Gold

**Affiliations:** 1 Maryland Psychiatric Research Center, Department of Psychiatry, School of Medicine, University of Maryland, Baltimore, Maryland, United States of America; 2 School of Public Health, Faculty of Health Sciences, Curtin University, Perth, Western Australia, Australia; 3 Curtin Health Innovation Research Institute—Biosciences, Curtin University, Perth, Western Australia, Australia; 4 Department of Psychology, University of New Mexico, Albuquerque, New Mexico, United States of America; 5 Department of Cognitive, Linguistic and Psychological Sciences, Brown University, Providence, Rhode Island, United States of America; 6 Department of Psychiatry and Brown Institute for Brain Science, Brown University, Providence, Rhode Island, United States of America; Benito Menni Complejo Asistencial en Salud Mental, SPAIN

## Abstract

The negative symptoms of schizophrenia (SZ) are associated with a pattern of reinforcement learning (RL) deficits likely related to degraded representations of reward values. However, the RL tasks used to date have required active responses to both reward and punishing stimuli. Pavlovian biases have been shown to affect performance on these tasks through invigoration of action to reward and inhibition of action to punishment, and may be partially responsible for the effects found in patients. Forty-five patients with schizophrenia and 30 demographically-matched controls completed a four-stimulus reinforcement learning task that crossed action (“Go” or “NoGo”) and the valence of the optimal outcome (reward or punishment-avoidance), such that all combinations of action and outcome valence were tested. Behaviour was modelled using a six-parameter RL model and EEG was simultaneously recorded. Patients demonstrated a reduction in Pavlovian performance bias that was evident in a reduced Go bias across the full group. In a subset of patients administered clozapine, the reduction in Pavlovian bias was enhanced. The reduction in Pavlovian bias in SZ patients was accompanied by feedback processing differences at the time of the P3a component. The reduced Pavlovian bias in patients is suggested to be due to reduced fidelity in the communication between striatal regions and frontal cortex. It may also partially account for previous findings of poorer “Go-learning” in schizophrenia where “Go” responses or Pavlovian consistent responses are required for optimal performance. An attenuated P3a component dynamic in patients is consistent with a view that deficits in operant learning are due to impairments in adaptively using feedback to update representations of stimulus value.

## Introduction

Patients with schizophrenia (SZ) have repeatedly shown performance impairments in trial-by-trial reinforcement learning (RL) tasks [1–5]. For example, Koch et al. [4] found significant impairments in patients learning from rewards and punishments for correct and incorrect responses respectively across both 80% and 100% contingency conditions. In a series of experiments we have found that these impairments are particularly prominent in those with high levels of negative symptoms [6–8]. Specifically, high negative symptom patients show impairments in learning to select the most advantageous response in order to gain rewards, but show relatively normal levels of performance when learning how to avoid punishments. Our work has suggested that these selective learning deficits are primarily related to degraded representations of prospective reward values of actions during choices [7]. Note however, that the tasks used in this set of experiments required participants to make active (or “Go”) responses to gain rewards and avoid punishments. Recent studies have shown that Pavlovian biases influence the performance on these types of operant learning tasks. Pavlovian bias refers to the linkage of affective states with action biases. In the present context, it is most notable that reward-predicting stimuli invigorate, and loss-predicting stimuli inhibit, active or Go responding [9,10]. Therefore, it is possible that reward learning deficits observed in patients can, at least in part, be explained by reductions in Pavlovian to instrumental transfer, rather than (or in addition to) degraded representations of instrumental action values.

Motivated action selection and learning are both strongly linked with striatal dopaminergic signals [11] Increased firing of dopamine neurons signal positive reward prediction errors (PEs) [12–15] and invigorates action, while reductions in dopamine firing signals negative reward PEs (outcomes that are worse than expected), which inhibit action. Thus reward-predicting cues can elicit positive dopamine signals that enhance “Go” responding, initiating an action in order to gain reward, whereas loss-predicting stimuli elicit reductions in dopamine that inhibit “Go” responding, resulting in a tendency to avoid making a response in order to avoid punishing outcomes. This provides a neural basis for an observed Pavlovian learning bias: i.e., it is far more difficult for subjects to learn to inhibit an action to obtain reward than it is to activate an action for a reward or to avoid a loss [9,10,16]. Indeed, pharmacological elevation of dopamine signalling is associated with enhanced striatal and midbrain representations of rewarding actions [17]. The possibility that alterations in dopamine signalling in SZ (either inherent to the illness or secondary to treatment with antipsychotics) might actually reduce Pavlovian biases and contribute to altered learning has not been addressed. Interestingly, such an account predicts that it should be possible to observe a performance advantage in patients when the withholding of a response leads to a reward, a theoretically interesting instance where an abnormality in an underlying process actually leads to a behavioural performance advantage.

In healthy volunteers, participants can exert cognitive control over Pavlovian biases to improve performance in Pavlovian-incongruent conditions (NoGo-to-reward and Go-to-avoid). Successful regulation of such biases are associated with activation in inferior frontal gyrus (IFG) and medial frontal cortex [9]. Medial prefrontal theta power has similarly been shown to be an electrophysiological index of cognitive control that increases in response to stimulus or response conflict [10,18–23], including overriding Pavlovian conflict [10]. There is a large literature documenting frontal cortical deficits in patients with SZ, including in the IFG [24–27]. From this perspective, one might thus expect the opposite pattern of results: patients would exhibit reduced ability to override Pavlovian conflict, thereby enhancing Pavlovian bias. This contrasts with the suggestion above, where reduced fidelity in dopaminergic signalling might attenuate Pavlovian bias by having a detrimental effect on valence-outcome pairing. Note, however, that if the factors driving the source of Pavlovian bias (putatively, striatal dopaminergic signals) are degraded, then there would be less need for cognitive control to override them.

It is necessary to also examine potential alterations in instrumental learning signals that could contribute to impaired learning of stimulus-response associations as likely modulators of behavioural patterns associated with Pavlovian Bias. Reward PEs and feedback processing signals are commonly observed within the context of the feedback-related negativity (FRN) that occurs approximately 250 ms post-feedback and which is hypothesised to be driven by phasic alterations of dopamine that affect instrumental learning ([28–30]; although see [31,32]). Recent data driven analyses have demonstrated additional later positive-going components that might contribute to attentional orienting and value updating in RL experiments. Specifically, Fischer & Ullsperger [33] reported that the signed PE signal extracted from a RL model correlated positively with the FRN (at 250 ms) representing PE processing. Moreover, the same PE signal correlated negatively with the feedback-elicited P3a and P3b, tracking the major deflections in the ERP (representing attentional orienting and contextual updating; [34]). Time-frequency decompositions of feedback activity have similarly shown larger frontal theta activity to loss feedback compared to win feedback [35–37], with medial frontal activity linked to the signed PE on a trial by trial basis.

In one of the few studies to have examined the FRN in SZ, Morris et al. [3] demonstrated a reduction in the FRN in SZ patients. However, a reduction was only present for the condition where responses mapped 100% to feedback, not for the 50% or 80% conditions. A follow-up study [38] and an independent investigation [39] similarly found no evidence for an FRN deficit in patients on an 80% contingent passive gambling task and a 50% contingent gambling task. Computational modelling of patients' ERN data in Morris et al. [38] indicated a deficit in the representation of response value rather than altered PE signalling. That is, patients appear to signal error feedback normally, but fail to use that feedback to adjust values to guide subsequent behaviour. If the ERN and the initial PE are relatively intact in patients, then it suggests that failures specific to guiding behaviour might emerge post-FRN, likely around the P3 region. The role of activity in this time period has yet to be explored in the SZ RL literature, an issue we address below.

We investigated whether Pavlovian biases exist to a similar extent in patients with SZ during a RL task that orthogonalises action requirements and outcome valence. Computational modelling was applied to trial by trial behaviour in order to capture and explain key features of the behavioural data, in particular Pavlovian bias and standard RL parameters with the influence of Pavlovian bias taken into account. Extracted trial-by-trial PEs obtained from the models were then correlated with feedback elicited EEG activity in order to relate key features of PE signalling with ERP measures, while controlling for both action and valence axes. Predicated on past experiments, we anticipated that patients (particularly those with high negative symptom burden) would show greater impairments compared to controls in reward “Go” learning compared to punishment “Go” learning. Two alternative hypotheses for enhanced or reduced Pavlovian bias in SZ were evaluated. The first hypothesis suggests an increase in Pavlovian bias due to degradation of prefrontal signals that would normally override such biases and reflected in a reduced theta response to conflict [10]. By contrast, the second hypothesis suggests that the source of the Pavlovian bias is reduced due to dysregulated dopamine activity, i.e., reduced valence-action linkage. Moreover, we predict intact early feedback-related EEG activity in patients (i.e., at the FRN), while later feedback-related activity associated with updating of value will be impaired. The latter could lead to impaired instrumental learning as well as reduced Pavlovian bias due to reduced updating of reward values. Above, we note that theta appears to signal two distinct aspects of RL: cognitive control over Pavlovian conflict and feedback valence. Our patient sample included an unusually high proportion of patients taking clozapine as their primary antipsychotic. Several studies have shown that baseline theta is elevated after transition to clozapine [40–44] and P3 amplitudes have also been shown to be elevated [42]. Therefore, clozapine status was included as a significant variable of interest due to theta activity being central to RL.

## Materials and Methods

### Ethics Statement

The study was approved by the University of Maryland Institutional Review Board. All participants gave written informed consent and the capacity to provide informed consent was documented by testing all participants on whether they could recall the demands of the study, the risks of taking part in the study, and demonstrated knowledge of their ability to withdraw from the study.

### Participants

Forty-eight participants with a diagnosis of SZ (N = 38) or schizoaffective disorder (N = 10; according to DSM-IV diagnostic criteria) and 32 controls were recruited for the experiment. Patients were clinically and pharmacologically (drug and dose) stable (> 4 weeks) outpatients from the Maryland Psychiatric Research Center or other nearby clinics. Controls were free from a lifetime history of SZ, other psychotic disorder, current Axis I disorder, drug dependence, neurological disorder, or cognitively impairing medical disorder, with no family history of psychosis in first-degree relatives. Controls were screened with the Structured Clinical Interview for DSM-IV [45,46]. One patient and one control were excluded for being unable to learn the easiest condition (Go-to-Win), defined as less than five correct responses. Three participants (2 SZ and 1 HC) were excluded for lack of deviance in responding, defined as making an extended run (> 40) of “Go” responses or “No-Go” responses. Forty trials covers close to a full block of persistent responding and it is known that for at least one participant this reflected gamepad malfunction. This left 45 SZs and 30 HCs for the behavioural analysis. Participants underwent detailed neuropsychological testing, see supplementary material (S1 File) for assessments reported on.

### Task

The task was derived from [9] and the EEG modification was derived from [10]. Four simple shape stimuli were presented 48 times each (total trials = 192) to participants in a pseudo-random order. Participants were instructed to respond by pressing a button (“Go”) or withhold responding (“NoGo”) to gain rewards (“Win”) or avoid punishments (“Avoid”). Stimuli were rewarded or punished at a probability of 0.8. Two stimuli were associated with reward (thumbs up image, reflecting monetary gain) and two stimuli were associated with punishment (thumbs down image, reflecting monetary loss). The alternative to reward or punishment was a neutral outcome (thumb to the side, no monetary change). Monetary gain or loss was set at $0.05 per trial. Action and valence were crossed, resulting in one of each of the four stimuli requiring “Go-to-Win”, “Go-to-Avoid”, “NoGo-to-Win” and “NoGo-to-Avoid” in order to achieve the best possible outcome. The stimulus presentation sequence and timings were as follows: a cross hair presented for 400–600 ms, the stimulus presented for 1000 ms, a no-response period presented for 250–2000 ms, a response window presented for 2500 ms indicated with an “O” for 1500 ms then a cross hair 1000 ms, finally feedback was presented for 2000 ms.

Participants were instructed that four images would be presented and they would have to decide on the best response to make (to press the button or to not press the button) by trial and error to win the most money possible. Participants were told that some images had a chance of winning money if they made the right decision and others had a chance of losing money if they made the wrong decision. Depending on the outcome associated with the correct response (achieving a gain or avoiding a loss) the best strategy to some stimuli will be to press the button while for other stimuli the best decision will be to withhold responding. Following instructions, participants were given a series of practice trials with unique stimuli to get accustomed to the task. They were instructed through a Go-to-Win block followed by a NoGo-to-Avoid block, explaining the response options and the probabilistic nature of the rewards or punishments. Following the explicit instruction block, participants underwent a second practice session with two stimuli (Go-to-Win and NoGo-to-Avoid) without instruction to ensure an understanding of both response options and the structure of the task. Before the onset of the main experimental training phase, participants were reminded that each image has one best decision option, to press, or not to press, and that it stays the same for the entire task. Finally, it was reinforced that all four combinations of stimulus-response pairings were possible.

### EEG recording and processing

EEG was recorded from a 32 channel Biosemi system. Data were recorded unreferenced with the ground at AFz using a sampling rate of 1024 Hz with 512 Hz hardware filters. Data were imported into EEGLAB [47], offline referenced to linked mastoids, filtered between 1 and 40 Hz and down-sampled to 256 Hz. Data were epoched from -1500 ms to 1500 ms around stimulus and feedback event codes. Epochs with large potential fluctuations were removed using EEGLAB's *pop_autorej* procedure (starting probability was set at 5 *SD* and the maximum % of epochs to reject per iteration was set at 5). The first pass cleaned EEG data underwent ICA using the AMICA algorithm [48] before further artifact rejection was applied based on detection of significant linear trends over the epoch in component space or abnormal component signal strength in both the 0–2 Hz range and the 20–40 Hz range [49]. Another round of ICA was repeated on the second pass cleaned data, which was used to subtract activity associated with eye blinks and eye movements. ERPs were baseline corrected to a 100 ms baseline. Time-frequency analysis using the time-frequency analysis function from within EEGLAB [47] was applied to the data at logarithmically spaced frequencies from 3 to 40 Hz. Time-frequency power was baseline corrected using the average of the power response from -300 to -200 ms.

### Modelling

Models were adapted from previous modelling efforts using this task [9,10]. The final model used in the analysis was a six parameter model that included reward sensitivity (*ρ*^*rew*^), punishment sensitivity (*ρ*^*pun*^), learning rate (*ε*), irreducible noise (*ξ*), go bias (*b)* and Pavlovian bias (*π*). Hierarchical Bayesian parameter estimation using Monte-Carlo Markov Chain was performed using Stan [50]. This procedure obtains full posterior distributions on each parameter (i.e. not just their best guess value but the uncertainty about those values), and this method was found to improve parameter recovery in simulation experiments relative to other approaches. See supplementary material (S1 File) for more detail.

### Statistical Analysis

Bayesian repeated measures ANOVA-style models and Bayesian style t-tests were used to analyse the behavioural data [51,52]. More detail on the models used are in the supplementary material (S1 File). The advantages of these models include: can incorporate a *t*-distribution to render the analysis robust to outliers and some distortions of the normal distribution; model unequal variances; shrinkage to improve estimation and control for multiple comparisons.

### Threshold Free Cluster Enhancement

Threshold Free Cluster Enhancement (TFCE) was developed to overcome problems associated with threshold selection for EEG data, that gives a fully parametric account of the functional brain response and the functional differences between groups [53,54]. TFCE was calculated according to the method in Mensen & Khatami [53] and Pernet et al. [54]. First, appropriate between-/within-subject t-statics or correlation coefficients were calculated for each time point and electrode for the ERP analysis, or time point and frequency for the time-frequency analyses. Clustering was applied using a thresholded 8 nearest neighbour approach in time and frequency space (for time-frequency analyses at channel FCz) or time and electrode space (for voltage analyses at Fz, F3, F4, FCz, Cz, C3, C4, Pz, P3, and P4). Violations of test assumptions and type I error rates were addressed using permutation statistics. See supplementary material (S1 File) for further details of the method and permutation testing.

### Single trial ERP and theta power relationship with PE

For the ERP traces, voltages on a trial by trial basis at all time points (i.e., from -200 to 1000 ms post-stimulus in 3.9 ms increments) were obtained for each individual. For each of the 307 time points, the estimated PEs obtained from from the RL model (from S1 File *ρ*^*rew|pun*^ * r–Q_*t-1*_[*a*_*t*_ *| s*_*t*_]; see e.g., [33]) were correlated with voltage using Spearman's rho. Spearman's rho coefficients underwent Fisher's r to z transform before entering into TFCE analysis and averaged for display. Similarly, for the relationship between PE and theta (4–8 Hz) power was averaged between 300 and 600 ms post-feedback onset for each trial. Bayesian linear mixed effects modelling using custom code calling Stan was used to regress theta power as a function of PE. Diagnostic group was included as an interacting factor with PE. Participants' intercepts and slopes were treated as random effects.

## Results

### Demographics

Demographic characteristics of the sample are presented in Table 1. Participants were well matched across age, sex, race and parental education. Patients were found to have lower education and cognitive ability compared with controls, as is usual for schizophrenia studies. We did not attempt to match participants on education as this would yield a non-representative higher education cohort of patients, as well as a non-representative low education cohort of controls.

**Table 1:** Demographics, neuropsychological performance and symptom ratings.

		HC (N = 30)			SZ (N = 45)				Cloz^-^ (N = 24)			Cloz^+^ (N = 21)		
	Mean		SD	Mean		SD	p	Mean		SD	Mean		SD	p
Age (yrs)	36.3		11.3	37.7		11.6	0.6	39.3		12.2	35.9		10.8	0.33
Gender (M | F)	20 | 10			32 | 13			0.88	17 | 7			15 | 6			1.00
Haloperidol Equivalent Dose				12.2		15.6		10.5		6.7	14.0		21.4	0.47
Number of APs (1 | 2+)				35 | 10				20 | 4			15 | 6			0.55
Education (yrs)	14.8		2.0	12.9		2.4	0.0004	12.3		2.0	13.5		2.6	0.10
Maternal Education (yrs)	14.0		2.3	14.2		2.8	0.85	13.7		2.6	14.8		2.9	0.20
Paternal Education (yrs)	13.8		2.6	14.5		2.8	0.33	13.3		2.3	15.9		2.7	0.002
Cognitive														
WASI IQ	110.5		10.6	95.0		16.7	<0.0001	96.0		15.4	93.9		18.3	0.68
WTAR	112.2		9.2	97.6		18.9	<0.0001	94.8		18.1	100.8		19.7	0.29
MD Working Memory	52.0		8.8	38.3		11.1	<0.0001	41.3		10.5	34.9		11.1	0.054
MD Processing Speed	53.9		10.6	38.8		10.8	<0.0001	39.4		10.1	38.1		11.6	0.70
MD Attention Vigilance	50.6		8.9	40.7		13.1	0.0002	44.0		11.8	36.9		13.8	0.072
MD Verbal Learning	49.1		8.9	36.9		8.6	<0.0001	40.3		8.9	33.1		6.5	0.003
MD Visual Learning	46.7		10.0	35.1		13.2	<0.0001	38.0		12.0	31.7		14.0	0.11
MD Reasoning	49.1		10.4	44.0		9.7	0.037	45.5		10.2	42.3		9.1	0.27
MD Social Cognition	52.3		9.8	38.0		11.1	<0.0001	39.0		9.5	36.8		12.8	0.52
MCT Overall	50.6		9.7	31.9		13.9	<0.0001	35.4		12.1	28.0		15.0	0.079
Symptom														
BPRS Affect				5.2		2.6		4.9		2.5	5.5		2.9	0.49
BPRS Negative Symptoms				5.8		2.7		5.4		2.4	6.3		3.0	0.27
BPRS Reality Distortion				7.3		3.6		6.2		2.8	8.7		4.0	0.022
BPRS Disorganisation				3.3		0.7		3.2		0.5	3.5		0.8	0.088
BPRS total				31.3		7.9		28.9		6.7	34.1		8.3	0.028
SANS Asociality Anhedonia				8.1		4.0		7.1		4.3	9.2		3.4	0.076
SANS Role Functioning				9.1		5.4		8.5		6.0	9.7		4.8	0.45
SANS Affective Blunting				8.9		6.2		7.9		6.1	10.1		6.4	0.24
SANS Alogia				1.0		1.5		0.6		1.2	1.4		1.8	0.087
SANS total				27.0		13.6		24.1		13.9	30.4		12.9	0.12

### Behavioural Performance

#### Accuracy and reaction time

**Fig 1:**
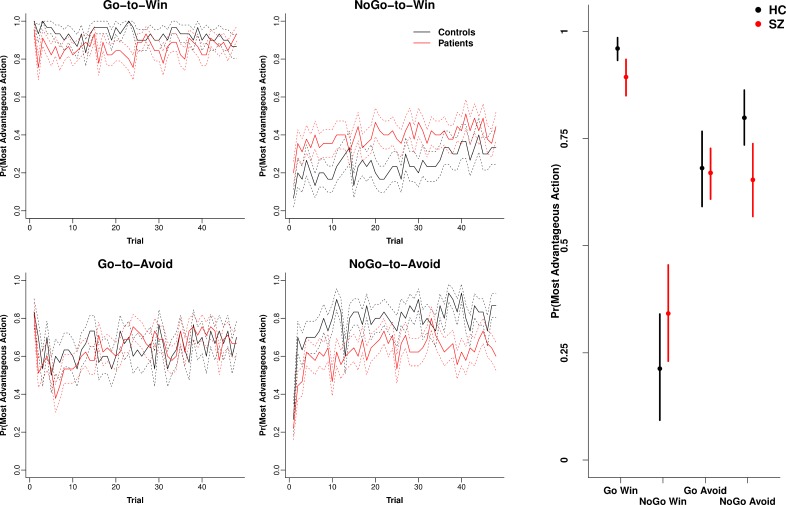
Performance accuracy. Left) Averaged performance across trials (± SE) for controls and patients with schizophrenia for each of the four conditions. Right) Means and 95% HDI intervals obtained from the posterior of the Bayesian ANOVA-style model for each of the four conditions. Credible reductions in patients compared to controls were observed for the “Go-to-Win” and “NoGo-to-Win” conditions. N = 45 patients and 30 controls.

Fig 1 (Left) illustrates the performance time course for each group and condition (mean ± SE). Performance followed the expected pattern based on the operation of Pavlovian biases with the greatest accuracy for Go-to-Win, followed by NoGo-to-Avoid and Go-to-Avoid, with poorest performance on NoGo-to-Win trials. Fig 1 (Right) presents the mean estimates (± 95% HDI) for summed performance accuracy across trials obtained from the Bayesian repeated measures ANOVA. Patients demonstrated credibly poorer accuracy on the two Pavlovian congruent conditions Go-to-Win and NoGo-to-Avoid relative to controls. In contrast, patients showed if anything *better* performance on the most difficult NoGo-to-Win condition, although this was not credibly different to controls. For a general overall comparison of the Bayesian approach with the Frequentist approach, we obtained a significant three way interaction between group, valence and action using repeated measures ANOVA (F[1, 73] = 9.5, p = 0.003), consistent with the pattern of differences between patients and controls for some stimuli found using the Bayesian method.

Mimicking the overall performance on win trials, patients displayed lower win-stay behaviour, compared to controls, on both Go-to-Win (win-stay probability controls = 0.97, patients = 0.90, effect size = -0.72, 95% HDI = -1.24, -0.20),and NoGo-to-Win (controls = 0.89, patients = 0.80, effect size = -0.58, 95% HDI = -1.04, -0.10). By contrast, there were no differences between patients and controls in lose-shift probability for Go-to-Avoid (overall lose-shift probability controls = 0.38, patients = 0.39, effect size = 0.05, 95% HDI = -0.44, 0.53) or NoGo-to-Avoid (controls = 0.33, patients = 0.35, effect size = 0.11, 95% HDI = -0.38, 0.59). Table 2 presents the results of correlation analyses between accuracy in each of the four conditions with cognitive performance and symptom measures. The strongest association was between cognitive ability in patients and performance on the Pavlovian consistent conditions (Go-to-Win and NoGo-to-Avoid).

**Table 2:** Correlation between Symptoms and neuropsychological performance with behavioural performance and modelled parameters.

			WASI Total IQ	MCT Overall	SANS Total	SANS Asociality-Anhedonia	SANS Role Function	SANS AA + RF	BPRS Total
Behavioural	Healthy	Go-to-Avoid	0.39 *	0.29					
Performance	Controls	NoGo-to-Avoid	0.28	0.25					
		Go-to-Win	0.22	0.22					
		NoGo-to-Win	0.06	-0.09					
		Pavlovian Performance Bias	-0.07	0.13					
	Schizophrenia	Go-to-Avoid	0.15	0.01	-0.26	-0.20	-0.37 *	-0.33 *	-0.02
	Patients	NoGo-to-Avoid	0.52 **	0.57 **	-0.21	-0.16	-0.18	-0.20	-0.10
		Go-to-Win	0.63 **	0.57 **	-0.29	-0.21	-0.24	-0.27	-0.19
		NoGo-to-Win	0.10	0.17	0.02	0.09	0.07	0.08	-0.13
		Pavlovian Performance Bias	0.33 *	0.31 *	-0.06	-0.069925422	-0.06	-0.04	-0.13
Modelling	Healthy	Model Fit (WAIC)	-0.48 **	-0.51 **					
	Controls	Reward Sensitivity	0.14	0.18					
		Punishment Sensitivity	0.43 *	0.31					
		Learning Rate	0.40 *	0.42 *					
		Irreducible Noise	0.23	0.06					
		Go Bias	0.23	0.28					
		Pavlovian Bias	-0.29	-0.14					
	Schizophrenia	Model Fit (WAIC)	-0.56 **	-0.45 **	0.25	0.20	0.24	0.25	0.01
	Patients	Reward Sensitivity ***ρ***^***rew***^	0.41 **	0.50 **	-0.14	-0.08	-0.02	-0.07	-0.16
		Punishment Sensitivity ***ρ***^***pun***^	0.44 **	0.44 **	-0.27	-0.22	-0.26	-0.29	-0.04
		Learning Rate ***ε***	0.48 **	0.52 **	-0.38 *	-0.29 *	-0.42 **	-0.44 **	-0.03
		Irreducible Noise ***ξ***	0.45 **	0.32 *	-0.15	-0.13	-0.22	-0.22	-0.18
		Go Bias ***b***	0.03	-0.25	-0.14	-0.22	-0.21	-0.25	0.05
		Pavlovian Bias ***π***	0.03	0.07	0.16	0.11	0.11	0.15	-0.10

* p < 0.05

** p < 0.01.

For the analysis of reaction time data, we included only the conditions requiring a response (Go-to-Win and Go-to-Avoid). The Bayesian repeated measures ANOVA indicated credible effects of diagnosis (SZ vs HC contrast = 24.3 ms, 95% HDI = 6.7, 42.3) and stimulus valence (Win vs Loss contrast = -23.9 ms, 95% HDI = -40.7, -7.4) indicating slower response times in patients and faster response times to positively valenced stimuli. There was not a credible diagnosis by valence interaction (contrast = 21.3 ms, 95% HDI = -9.6, 54.7). A comparative Frequentist approach only indicated a significant main effect of valence (F[1, 73] = 8.7, p = 0.004), with the win condition yielding faster reaction times than the avoid condition, but no main effect of diagnosis (F[1, 73] = 0.62, p = 0.43) nor was there a diagnosis by outcome interaction (F[1, 73] = 1.4, p = 0.25).

#### Pavlovian Bias

**Fig 2:**
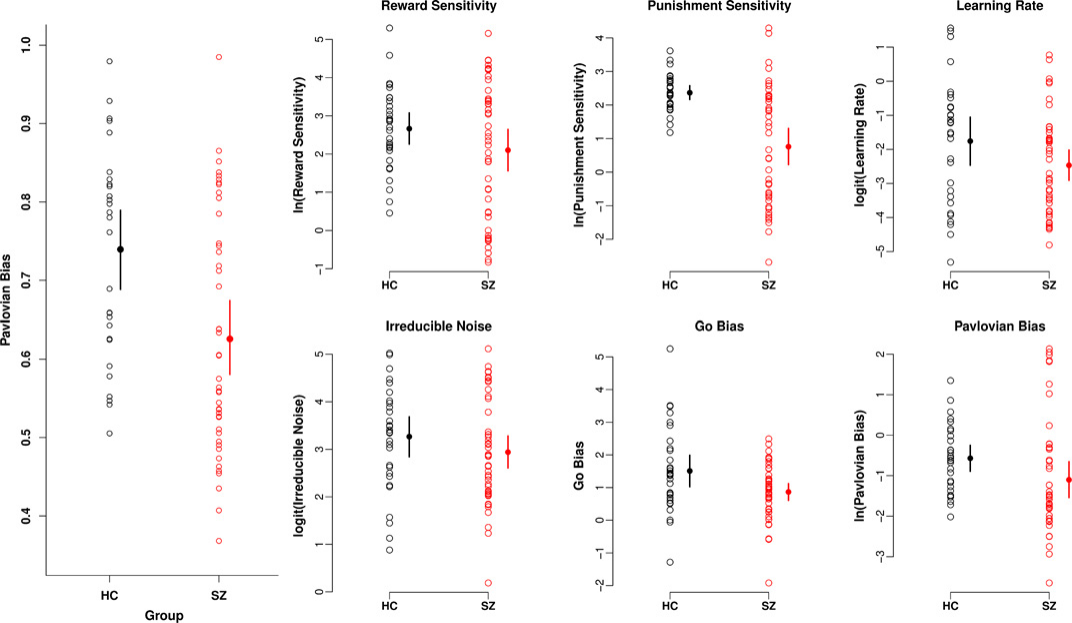
Pavlovian bias and modelled parameters. Left) Pavlovian performance bias calculated from the behavioural data contrasting reward based invigoration and punishment based-suppression. A larger value indicates greater Pavlovian bias. Right) Parameters extracted from the final reinforcement learning model used to fit the data. Means and 95% HDI of the posterior are presented obtained from a robust Bayesian t-test.

We calculated a single measure of Pavlovian performance bias by averaging reward-based invigoration and punishment based suppression (see Methods; [10]). Fig 2 (Left) illustrates the mean (+ 95% HDI) for each group (obtained from a robust Bayesian t test), indicating less Pavlovian bias in patients (mean = 0.63, 95% HDI = 0.58, 0.67) compared to controls (mean = 0.74, 95% HDI = 0.69, 0.79; effect size = 0.78, 95% HDI = 0.30, 1.28). Pavlovian bias was correlated with cognitive ability in patients, consistent with the positive correlation reported above between cognitive ability and performance on the two Pavlovian consistent conditions.

#### Modelling

Table 3 presents the model fits (using the Widely Applicable Information Criteria [WAIC] and the Bayesian Information Criteria [BIC]) and mean parameter estimates (+ SD; parameters presented on the sampled scale) for each of the RL models fitted. The final six parameter model that incorporated reward sensitivity (*ρ*^*rew*^), punishment sensitivity (*ρ*^*pun*^), learning rate (*ε*), irreducible noise (*ξ*), go bias (*b)* and Pavlovian bias (*π*) was used to extract estimates for each participant. S1 Fig presents the group averages of the simulated output obtained from each individual's fitted parameters using the full posterior; the output here can be contrasted with Fig 1 showing good re-creation of the data using the model. Fig 2 displays the modelled coefficients and their means (+ 95% HDI) by group. Punishment sensitivity ***ρ***^***pun***^ was the most strongly reduced parameter in the patient group (effect size = 1.23, 95% HDI = 0.73, 1.74). The go bias parameter ***b*** was also reduced in patients relative to controls (effect size = 0.60, 95% HDI = 0.090, 1.13). While the Pavlovian bias parameter ***π*** strongly correlated with the behavioural measure of Pavlovian bias (Spearman's rho = 0.76, p < 0.0001) which was credibly reduced in patients (see above), there was not a credible reduction of the parameter ***π*** in the patient group (effect size = 0.46, 95% HDI = -0.030, 0.95).

**Table 3:** Modelling WAIC and overall fit.

	M1	M2	M3	M4	M5	M6 Controls	M6 Patients
WAIC	15143	15090	13766	13266	11681	11666	
BIC	15057	14985	13606	13033	11398	11393	
ln(Feedback Sensitivity)	1.5 (1.05)	2.3 (1.1)	2.1 (1.2)				
ln(Reward Sensitivity)				2.8 (1.4)	2.6 (1.9)	2.7 (1.6)	2.0 (2.3)
ln(Punishment Sensitivity)				2.3 (1.7)	1.7 (1.5)	2.4 (0.78)	0.87 (2.0)
logit(Learning Rate)	-2.3 **(**2.6**)**	-2.3 **(**2.7**)**	-2.3 **(**2.7**)**	-2.1 **(**2.9**)**	-2.3 **(**2.2**)**	-1.7 **(**2.1**)**	-2.4 **(**2.0**)**
logit(Irreducible Noise)		2.7 (2.3)	3.0 (2.1)	2.5 (2.0)	2.9 (1.8)	3.2 (1.6)	3.0 (1.7)
Go Bias			0.72 (1.0)	0.86 (1.2)	1.2 (1.2)	1.5 (1.6)	0.87 (1.0)
ln(Pavlovian Bias)					-0.70 **(**1.3**)**	-0.55 **(**1.0**)**	-1.0 **(**1.8**)**

The bottom half of Table 2 details the correlations (Spearman's rho) between cognitive ability and symptom ratings with each of the modelled paameters. Higher negative symptoms were associated with a lower learning rate parameter *ε*, including SANS total, SANS Anhedonia & Asociality, SANS Role Functioning and the combined Asociality & Anhedonia/Role functioning. In addition, cognitive ability was correlated with model fit in both patients and controls, with better model fits associated with higher cognitive ability. Cognitive ability was also positively correlated higher learning rates and reward/punishment sensitivities, which was most notable in the patient group.

#### Effect of clozapine on behaviour and modelled parameters

**Fig 3:**
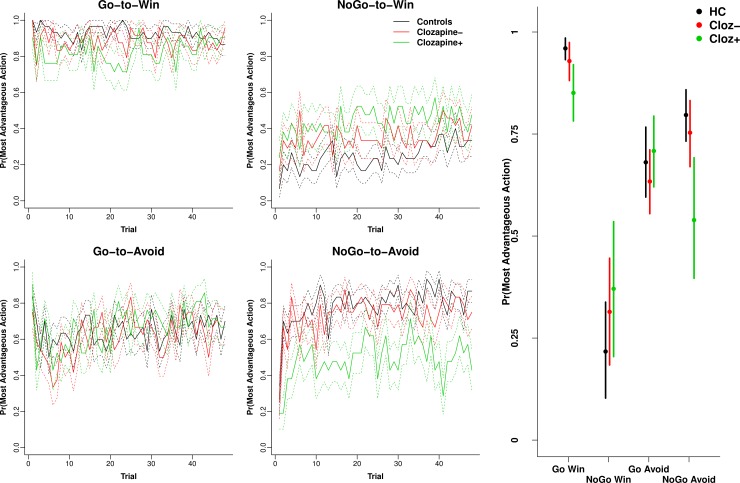
Clozapine Performance accuracy. Left) Averaged performance across trials (± SE) for controls and patients broken down by clozapine status for each of the four conditions. Right) Means and 95% HDI intervals obtained from the posterior of the Bayesian ANOVA-style model for each of the four conditions. Clozapine enhanced the patient control differences depicted in Fig 1.

The Cloz^+^ group had higher paternal education, lower verbal learning, more BPRS reality distortion and BPRS total symptoms (Table 1). Cloz^+^ patients demonstrated amplified performance deficits on the Pavlovian congruent conditions Go-to-Win and NoGo-to-Avoid (Fig 3 Left and Right), but there was little difference on the NoGo-to-Win or Go-to-Avoid conditions between the clozapine groups. This led to a magnified reduction of Pavlovian bias in the Cloz^+^ group (S4 Fig Left; mean = 0.56, 95% HDI = 0.49, 0.62) compared to Cloz^-^ (mean = 0.68, 95% HDI = 0.62, 0.75; effect size = 0.90 (95% HDI = 0.23, 1.6) and controls (mean = 0.74, 95% HDI = 0.69, 0.79; effect size = 1.32, 95% HDI = 0.59, 2.02). Cloz^+^ patients were also fitted with a lower Pavlovian bias *π* compared to Cloz^-^ and controls (S2 Fig Right). An ANCOVA including symptoms (BPRS RD, SANS AA, SANS Alogia or SANS total) or general cognitive ability (WASI IQ) did not substantially diminish the reported association between clozapine and Pavlovian bias.

### EEG

#### Feedback: Loss versus win

**Fig 4:**
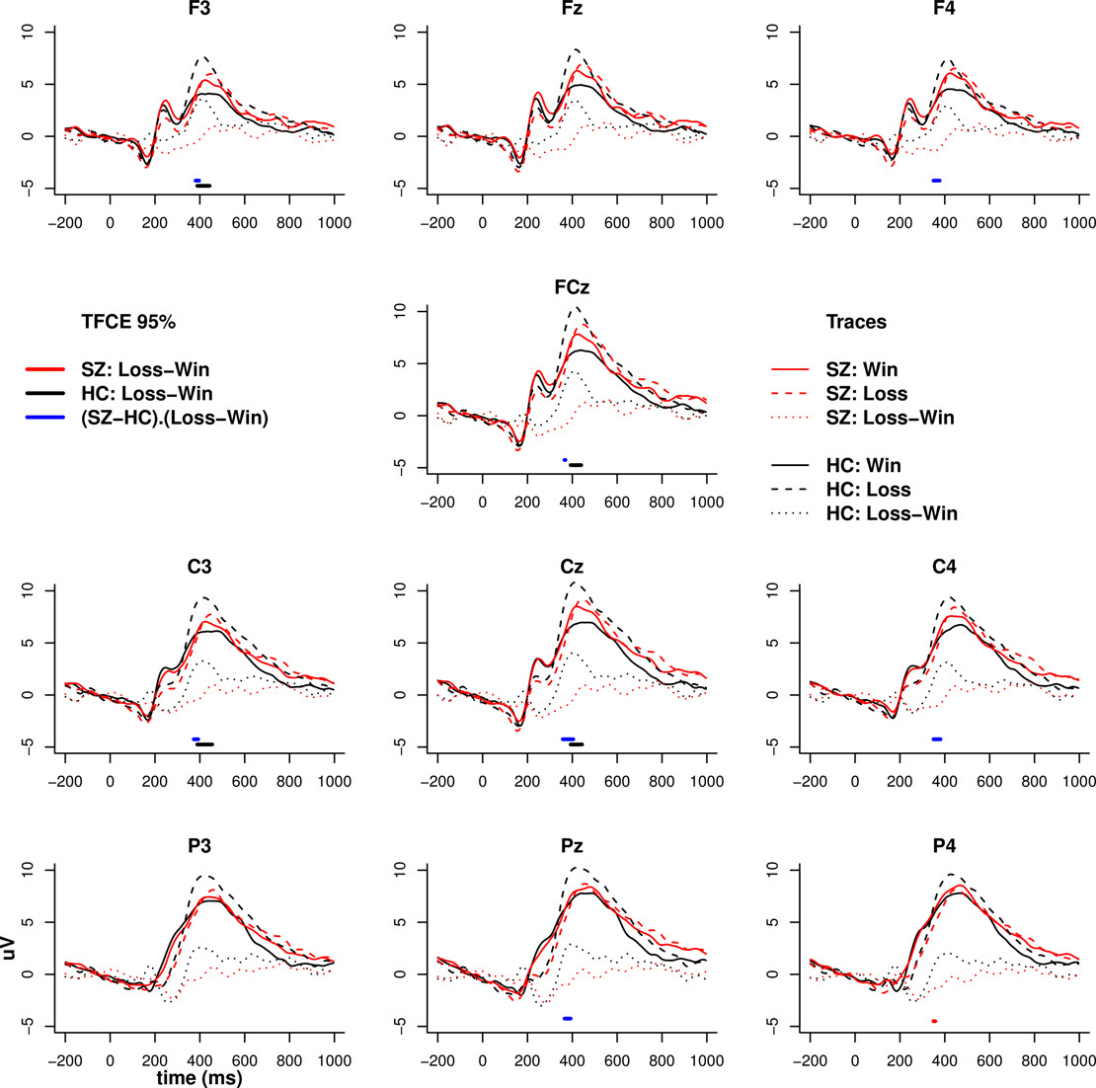
ERP to feedback. ERP to wins (thumbs up; solid lines), losses (thumbs down; dashed lines) and their contrast (dotted lines) for patients and controls. Solid horizontal bars at the bottom of each trace represent Threshold Free Cluster Enhancement (TFCE) significance values at 0.05 obtained from permutation testing.

**Fig 5:**
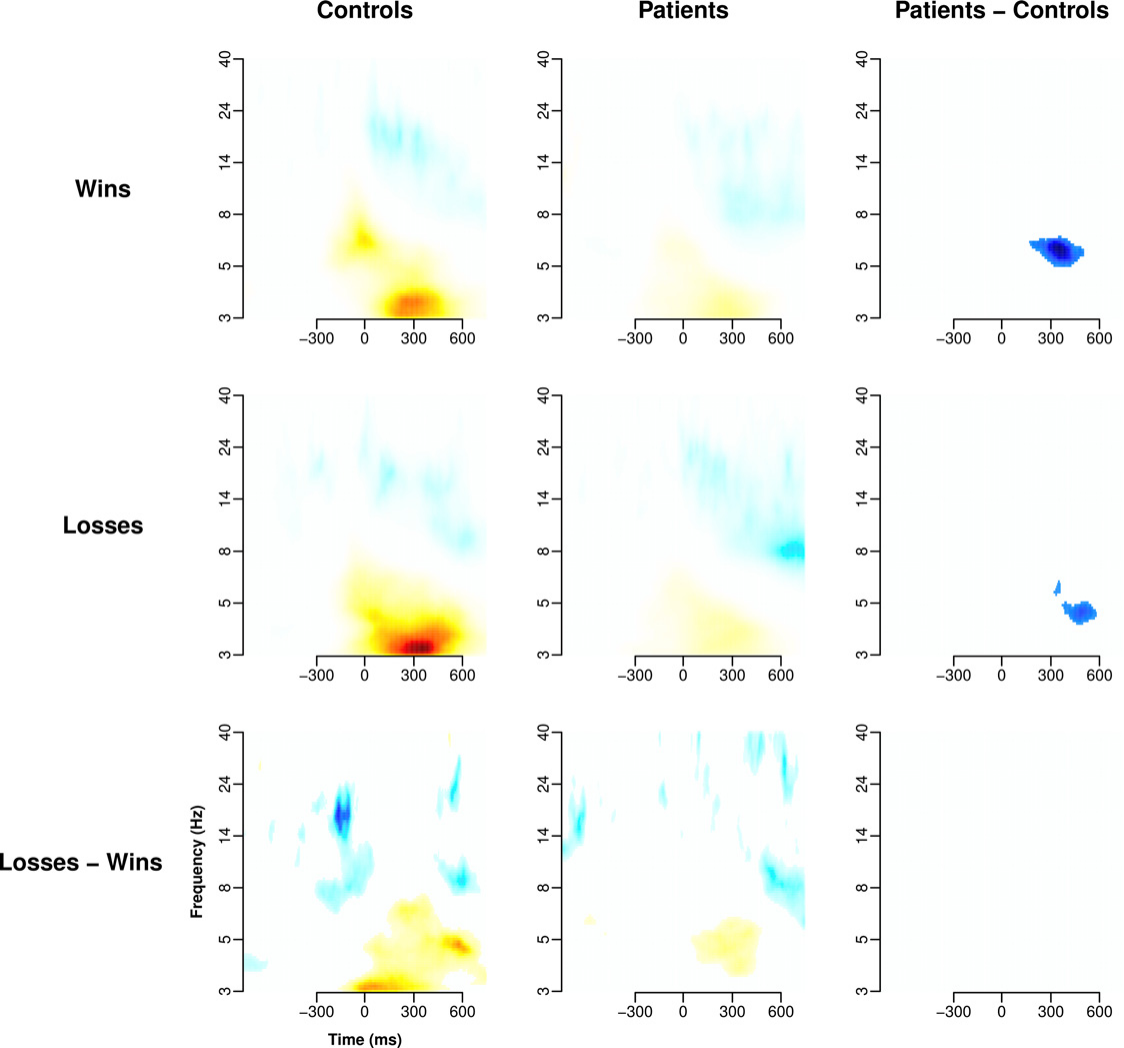
Time-frequency response to feedback. TFCE filtered time-frequency maps for wins, losses and their contrasts. Time-frequency analysis was conducted on the average of the three central midline electrodes Fz, FCz, and Cz. Colours are arbitrary, but symmetrical, mappings derived from the TFCE analysis scaled for best contrast. White spaces represent no significant contrast.

Figs 4 and 5 illustrates the feedback-locked ERP and time-frequency maps (after TFCE) for punishment and reward feedback. Feedback ERP differences between patients and controls emerged around 400 ms post-feedback, with controls showing a differential response to win and loss stimuli that was not evident in patients (significant feedback valence by diagnostic group interaction). The time-frequency analysis mirrored the ERP analysis in controls who demonstrated a more pronounced increase in low theta/high delta frontal midline power (which strongly reflects P3 amplitude) to loss compared to win. Compared to controls, patients demonstrated a reduction in late (~ 500 ms) theta (4–7 Hz) power to both win and loss stimuli. Unlike the ERP analysis, there was no interaction between feedback valence and diagnostic group.

#### Relationship between EEG feedback and PE

**Fig 6:**
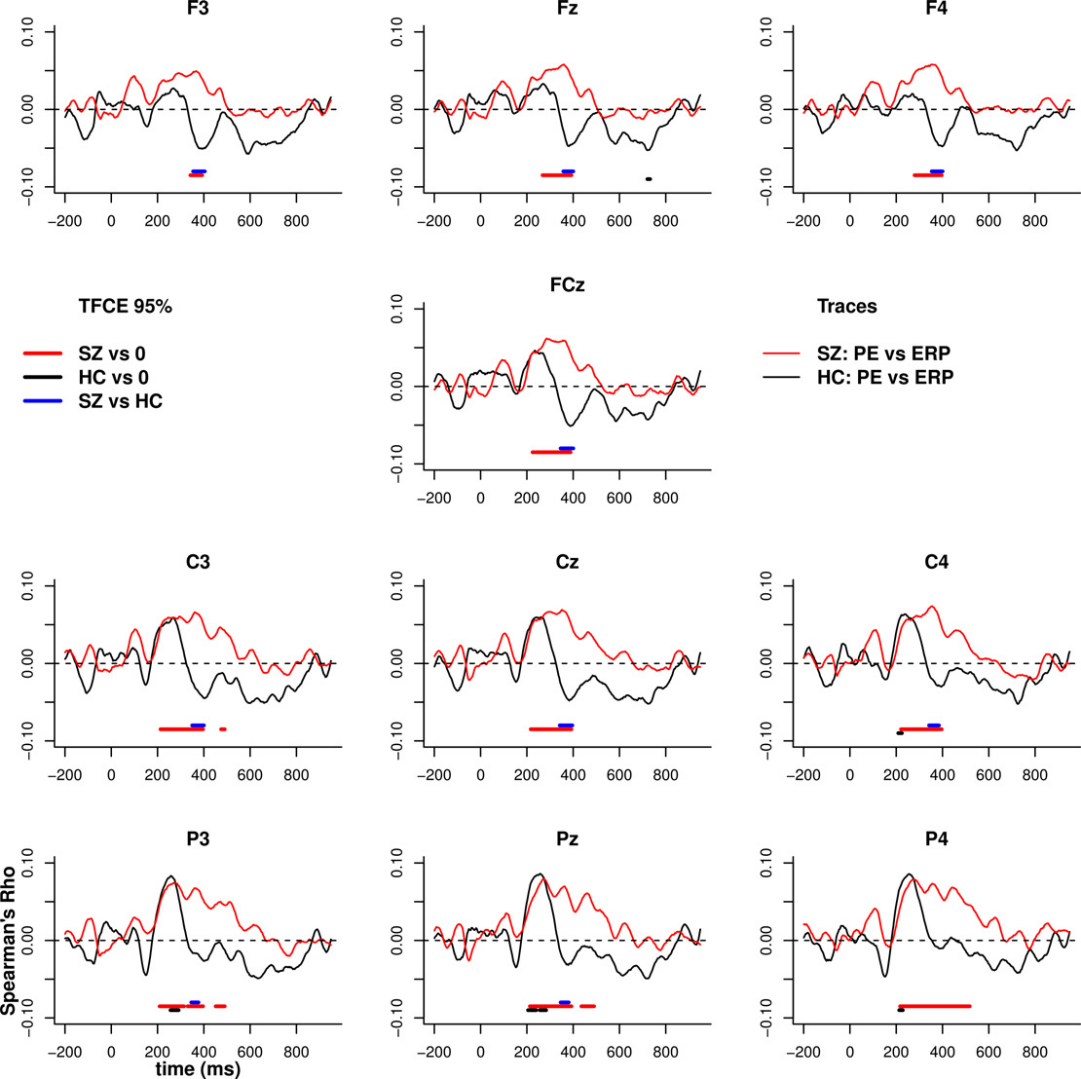
Relationship between PE and voltage. Spearman's correlation between voltage and the estimate of prediction error (PE) obtained from the reinforcement learning model. Correlations between PE and voltage were calculated for each person across all trials at each time point then group averaged. Solid horizontal bars indicated TFCE significance at 0.05. The relationship between PE and voltage is significant for both phases using a directed contrast.

Fig 6 illustrates the trial by trial voltage correlation with PE across the full epoch. Control participants demonstrated the usual positive then negative correlation between voltage and PE, corresponding with the polarity reversal in the theta-band sequence underlying the FRN and P3 components (significant at uncorrected alpha of 0.05 and consistent with previous reports; [33]). Interestingly, patients were characterised by an earlier more frontal and prolonged negative association between PE and voltage, beginning from the FRN and continuing throughout the P3. The relationship between voltage with positive PE and negative PE are presented in S3 and S4 Figs.

The relationship between PE and theta power (3–6 Hz at 300–600 ms) was estimated using Bayesian mixed-effects regression. Greater theta power co-varied negatively with PEs in controls (mean estimate = -0.17, 95% HDI = -0.26, -0.08), and this relationship was credibly flatter in the full patient group (mean estimate = -0.042, 95% HDI = -0.13, 0.043; contrast estimate SZ-HC = 0.13, 95% HDI = 0.003, 0.25). Thus reduced Pavlovian bias in patients was accompanied by altered neural signalling of PEs.

#### Conflict induced theta

We were unable to replicate the association between Pavlovian conflict and stimulus-locked frontal theta presented in [10]. Follow up analysis restricted to a subset of higher performing participants (performance on NoGo-to-Win > 10 correct, N = 12 controls and N = 23 patients) also failed to find an effect of Pavlovian conflict on theta.

#### Effect of clozapine on the EEG

Consistent with previous reports and justifying separating out the patients taking clozapine, robust Bayesian t-tests indicated credibly higher baseline theta (averaged between 200–300 ms pre-stimulus and 4–8 Hz) in patients administered clozapine (mean = 5.41, 95% HDI = 5.24, 5.58) compared to those on other antipsychotics (mean = 4.82, 95% HDI = 4.69, 4.96; effect size = 1.76, 95% HDI = 0.90, 2.64) and controls (mean = 4.75, 95% HDI = 4.67, 4.83; effect size = 2.3, 95% HDI = 1.35, 3.25).

S5 and S6 Figs present the EEG analyses with groups separated by clozapine status. The most notable difference was a greater reduction in feedback theta power in Cloz^+^ compared to controls, although this was not significantly different comparing Cloz^+^ to Cloz^-^ patients. However, a more targeted analysis at the peak of the theta feedback using the Bayesian mixed-effects regression described above relating trial-by-trial theta power with PE indicated a credible reduction in feedback-elicited theta power in Cloz^+^ (mean = 0.28, 95% HDI = -0.16, 0.72) relative to the Cloz^-^ group (mean = 1.39, 95% HDI = 1.04, 1.74) and controls (mean = 1.91, 95% HDI = 1.57, 2.25). There were no other credible or significant differences between the two subsets of patients. Overall, Cloz^+^ patients showed reduced Pavlovian bias in behavioural measures and model parameters, and this was also accompanied by altered EEG signals associated with PE.

## Discussion

We found a reduction in the behavioural evaluation of Pavlovian performance bias in patients with SZ, which was mostly manifest in terms of performance reductions in the two Pavlovian congruent conditions: Go-to-Win and NoGo-to-Avoid, with non-significant enhancements in the most-difficult incongruent NoGo-to-Win condition. However, an overall SZ effect on the modelled Pavlovian performance bias was only seen in patients taking clozapine. Reductions in Pavlovian biases were accompanied by alterations in neural signalling of feedback, including: reduced differentiation between loss and gain feedback-locked ERPs post-FRN, an altered relationship between voltage and PE in the SZ group, and a similarly altered relationship between theta power and PE. Computational modelling of the trial-by-trial behaviour suggested reduced go bias in patients that may have in part driven the reduction in behavioural Pavlovian bias in patients across the group. After examining the group of patients taking clozapine, we found that the behavioural effects in patients were enhanced in the clozapine group, including reduced Pavlovian bias and a reduction in the modelled Pavlovian bias parameter.

### Reduced Pavlovian bias

Reduced Pavlovian bias in patients could potentially be considered an enhancement of function because previous research has shown that the ability to over-ride this bias is strongly dependent on frontal inhibitory functions, similar to those used for executive functioning. Moreover, individuals who are able to overcome this bias and more strongly recruit frontal cortex tend to perform better at this specific RL task [9]. However, it seems unlikely that reduced Pavlovian bias in patients reflects an overriding by the frontal cortex of the action-valence link [9,10]. There is extant literature detailing impairments in frontal processes and neurophysiology associated with the overriding cognitive conflict patients, including in the critical conflict override region of the IFG [24–26]. We unfortunately found no conflict-related theta signal in prefrontal cortex during Pavlovian conflict as we had seen previously in young healthy subjects [10], which would have provided a direct assessment of this hypothesis. Nevertheless, we think the most likely explanation for reduced Pavlovian bias in SZ is a reduction of the striatal dopamine-driven mechanisms that normally fuel the bias in the first place, e.g., with antipsychotic medication or innate noise in the dopamine system [55]. Similarly, the bias could result from impairments in communication between the striatum and frontal cortex. Indeed, several studies have shown a reduction in connectivity between striatal and frontal regions during reward processing and working memory performance in patients with SZ [56–58] including in unmedicated patients during both reward and loss-avoidance [56]. Given that it was the Pavlovian consistent conditions that were the most affected behaviourally in patients (as well as a modest performance enhancement in the NoGo-to-Win Pavlovian conflict condition, thereby levelling out the performance between Pavlovian consistent and conflict conditions), this finding is consistent with altered information flow from dopamine signalled PEs to evidence weighing frontal cortical areas. This conforms with our ERP findings discussed below.

Modelling further suggested that some of the reduction in the behaviourally determined Pavlovian bias may have been due to a reduction in Go bias. Go bias is driven primarily by behaviour during the earliest trials of the task. Go bias reductions could potentially reflect the performance equalisation seen during the combined Win trials, particularly as the NoGo-to-Win condition benefits from inaction and is the most difficult condition to learn. It is possible that reductions in the Go bias parameter are a consequence of antipsychotic medications, e.g., via a reduction in dopamine signalling, a reduction in psychomotor activation or by impairing the attribution of incentive value to reward predicting stimuli (e.g., [59–62]). However, deficits in reward learning and striatal signalling have been demonstrated in non-medicated patients [63,64], suggesting an inherent processing alteration in SZ.

### Clozapine effects on behaviour

It is interesting to note that many of the behavioural effects, including both behavioural and modelled Pavlovian bias, were amplified in patients taking clozapine. Patients on clozapine also showed large increases in baseline theta power together with altered neural signalling of PEs. The field still lacks a precise understanding of the pharmacological differences between clozapine and other antipsychotics making it difficult to draw firm conclusions about how clozapine's pharmacology gives rise to these effects. Several candidate mechanisms for clozapine's unique status have been proposed, including increased serotonergic affinity [65–67], faster D_2_ dissociation [68], regulation of the glutamate system [69], and activity of its metabolite (NDMC) [70,71]. For example, we discuss below the influence of serotonin depletion on punishment or error driven learning [72] relevant for findings in the NoGo-to-Avoid condition that shows one of the largest effects of clozapine. Alternatively, above we suggest that a reduction in effective dopamine signalling or communication between frontal and striatal regions could explain the poor performance on the Pavlovian congruent conditions. However, given that this communication impairment is present in unmedicated patients and that clozapine has relatively less or similar affinity at dopamine receptors as other antipsychotics, it may instead reflect the likelihood that patients on clozapine tend to be a distinct sub-type of patient. For example, patients on clozapine generally have more treatment resistant symptoms that may not be associated with the same presynaptic dopamine hyperactivity seen in treatment responsive patients [73]. Moreover, the clozapine administered patients may possess a different cognitive and symptom profile (as partially described in Table 1). Replication of the influence of clozapine on reinforcement learning tasks may yield further insights into the unique effectiveness of this antipsychotic.

### Negative symptoms and reinforcement learning

We hypothesised a reduction in reward learning and reward sensitivity in patients that would be amplified in those with a high negative symptom burden in addition to a maintenance of punishment learning. Using the most equivalent comparison with previous findings by focussing exclusively on the Go conditions, we did find poorer performance compared to controls on Go-to-Win trials and equivalent performance on Go-to-Avoid trials, consistent with previous reports [6–8]. There was also a weak correlation between negative symptoms and performance on Go-to-Win trials, but this was not significant for the previously identified Anhedonia-Asociality measure (although the effect was in the expected direction). Somewhat surprisingly, we found a substantial reduction in Punishment sensitivity that appeared to be driven by poor performance on the Pavlovian consistent NoGo-to-Avoid condition. On the surface, this appears inconsistent with previous findings from our lab of selective deficits in reward learning with preserved punishment-driven learning. However, the punishment-driven learning for which there is the greatest evidence of preservation in SZ is of a gradual/procedural nature, involving incremental adjustments in stimulus-response association strength across many trials. Previous evidence linking RL performance to negative symptoms was largely based on transfer phase performance and not trial-to-trial learning. There is a large literature on reduced sensitivity to error feedback on a trial-to-trial basis in patients, leading to impairments in the ability to make rapid modifications to behaviour [74,75]. Indeed, we have recently observed a similar tendency to perseverate in the context of a task designed to investigate the contribution of working memory to RL [76]. In the model described here, punishment sensitivity directly impacts behavioural adjustments on the following trial. Reduced punishment sensitivity may also be a consequence of the serotonergic antagonist profile of most antipsychotics, as well as a general failure to respond to losses in order to rapidly adjust behaviour, as has been documented previously (e.g., [76]). In a similar task to that used in the present study, Helmbold et al. [72] found reduced neural sensitivity (assessed with fMRI) to punishment after acute tryptophan depletion, particularly during the NoGo-to-Avoid condition. Indeed, reduced punishment sensitivity and performance on the NoGo-to-Avoid conditions were amplified in participants taking clozapine which possesses particularly strong serotonergic affinity.

### Feedback ERP and time-frequency effects

Several converging lines of evidence indicated an interesting dissociation between patients and controls during feedback processing. Consistent with earlier reports, we did not find any significant differences between patients and controls at the classic FRN latency [3,38,39], suggesting that the earliest component of feedback processing that is associated with signalling PEs is relatively intact in patients. Striking differences emerged around 400 ms post-feedback, with controls demonstrating an enhanced positivity to loss feedback compared to reward and this was differentially reduced in patients. Previous research has identified a similar lack of loss-evoked positivity in patients relative to controls, as shown in Fig 3 of [39]. However, this was not analysed or discussed by the authors. Further single-trial analyses indicated that the relationship between PE and voltage/theta was altered compared to controls at this later processing stage.

The later feedback processing differences between patients and controls occurred in a temporal and spatial pattern most consistent with the P3a response to feedback. The P3a is typically linked with attention orienting [34] and, in a feedback context, is suggested to signal salience and drive attention towards the stimulus [31]. A more posterior system then becomes involved, tied to accumulation of evidence in order to make a decision [77–79] as well as updating stimulus value, indexed by the P3b [31]. While the P3b association with PE was noticeable in controls, the relationship was small and not statistically significant, possibly because this relationship is relatively dampened compared to the relationship between PE and earlier feedback processing components [33]. Alternatively, it may have been because there were many trials that did not require a response, which significantly modulates the P3b [78,79]. Feedback processing disruptions that occur at a later stage than the typical PE signal (the FRN) are consistent with evidence for intact model free learning in patients, while adding to increasing evidence for higher order model-based learning deficits [7,76]. Further evidence showing a clear disruption of the P3b relationship with the PE signal could strengthen this interpretation.

### Limitations

A possible reason for the lack of robust associations with negative symptoms in the present study is due to failure to recruit enough patients with very severe negative symptoms. Previous reports demonstrating this relationship recruited a greater number of participants with high SANS scores, enhancing the ability to find a relationship with negative symptoms [6–8]. Another limitation relates to being unable to replicate the conflict-evoked theta response seen in previously in Cavanagh et al. [10]. This may have been due to recruiting an older and more heterogeneous group compared to undergraduate university students used in previous conflict studies yielding a poorer signal to noise ratio of the ERP and time-frequency analysis. Alternatively, the presence of this effect should be contingent on a sub-group of participants learning the task with a rule-driven or “model-based” strategy, which may not have been present even amongst the highest performers.

## Conclusions

We found a reduction in Pavlovian bias in the entire patient sample that was amplified in patients on clozapine. We argue that the most likely explanation for this attenuation is a reduction of striatal dopamine-driven mechanisms that link feedback with behaviour. We suspect that this abnormal dopaminergic modulation of the striatum is more likely the result of disrupted communication between the striatum and frontal cortex, as opposed to better override of bias by the IFG. Furthermore, consistent with previous work showing that higher order deficits provide the most parsimonious explanation for RL performance in patients, electrophysiological evidence for feedback processing abnormalities in SZ was most notable post-FRN, during the P3a that indexes attentional resource allocation.

## Supporting Information

S1 Fig Simulated group accuracy from the six parameter model. Left) Behaviour was simulated by extracting each participant's modelled parameters from the posterior of the model fit and averaging simulated behavioural performance over the full posterior. Right) Total accuracy simulated for each participant (averaged over the full posterior) was used to generate means (+ 2 * SE) for the four conditions for each group.
(TIF)

S2 Fig Pavlovian bias and modelled parameters by clozapine status. Left) Pavlovian performance bias calculated from the behavioural data. Larger values indicate greater Pavlovian bias. Right) Parameters extracted from the final reinforcement learning model used to fit the data. Means and 95% HDI of the posterior are presented obtained from a robust Bayesian t-test.
(TIF)

S3 Fig Relationship between positive PE and voltage. Spearman's correlation between voltage and +ve PE. Correlations between +ve PE and voltage were calculated for each person across all trials at each time point then group averaged.
(TIF)

S4 Fig Relationship between negative PE and voltage. Spearman's correlation between voltage and -ve PE. Correlations between PE and voltage were calculated for each person across all trials at each time point then group averaged.
(TIF)

S5 Fig ERP to feedback by clozapine status. ERP to wins (thumbs up; solid lines), losses (thumbs down; dashed lines) and their contrast (dotted lines) for controls and patients by clozapine status. TFCE significance indicated by the solid horizontal bars at the bottom of each trace.
(TIF)

S6 Fig Time-frequency response to feedback by clozapine status. TFCE filtered time-frequency maps for wins, losses and their contrasts on the average of the three central midline electrodes Fz, FCz, and Cz. Colours are arbitrary, but symmetrical, mappings derived from the TFCE analysis scaled for best contrast.
(TIF)

S1 File Extended methods. Includes information on RL modelling, TFCE, neuropsychological assessment methods and Bayesian analysis details.
(DOC)
