# Supplementary figures and images for: Reduction of Pavlovian Bias in Schizophrenia: Enhanced Effects in Clozapine-Administered Patients
Source: PLoS One. 2016 Apr 4;11(4):e0152781. doi: 10.1371/journal.pone.0152781 (PMC4833478; doi:10.1371/journal.pone.0152781)

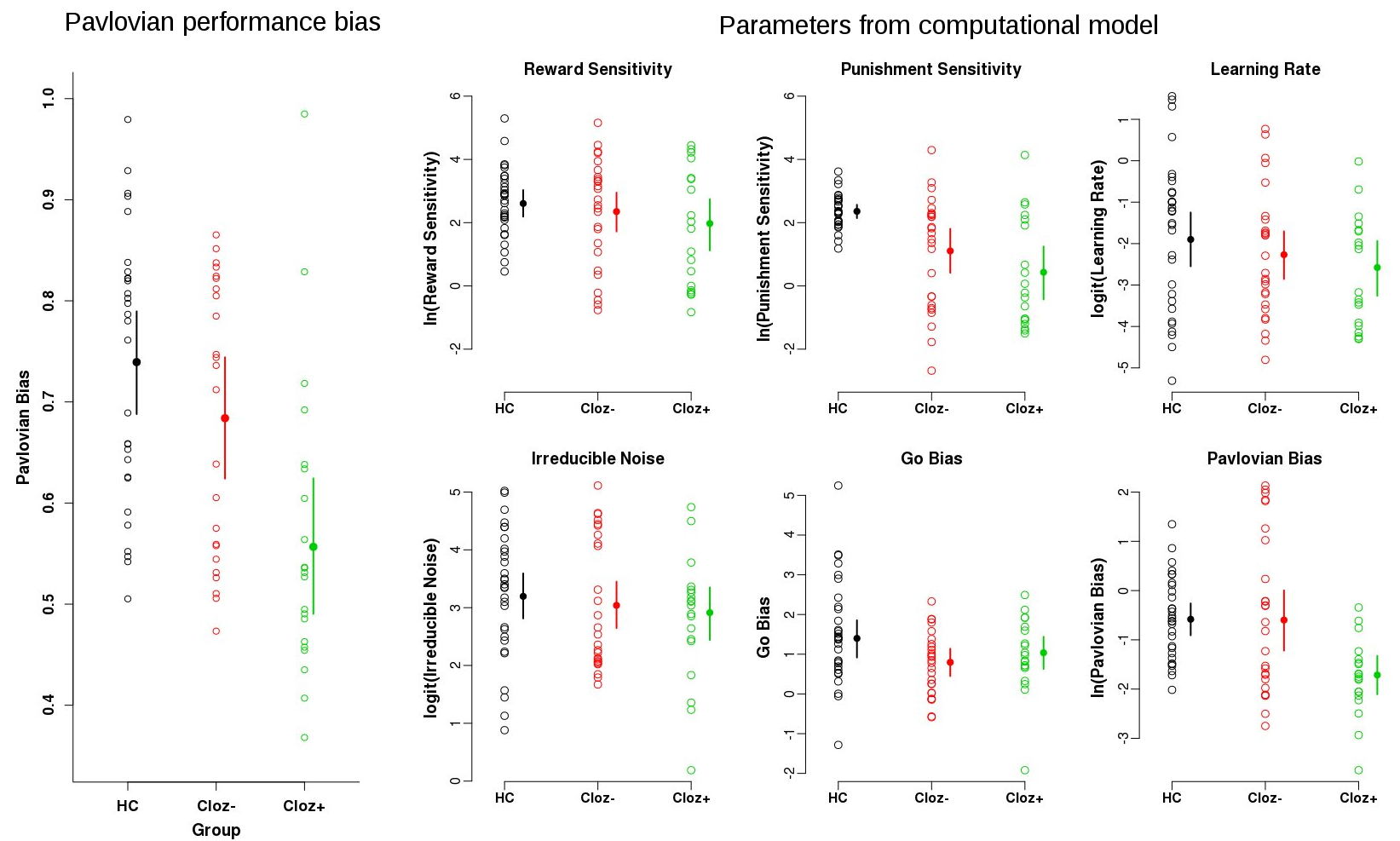

Supplement: S2 Fig — Left) Pavlovian performance bias calculated from the behavioural data. Larger values indicate greater Pavlovian bias. Right) Parameters extracted from the final reinforcement learning model used to fit the data. Means and 95% HDI of the posterior are presented obtained from a robust Bayesian t-test. (TIF) [file pone.0152781.s002.tif]

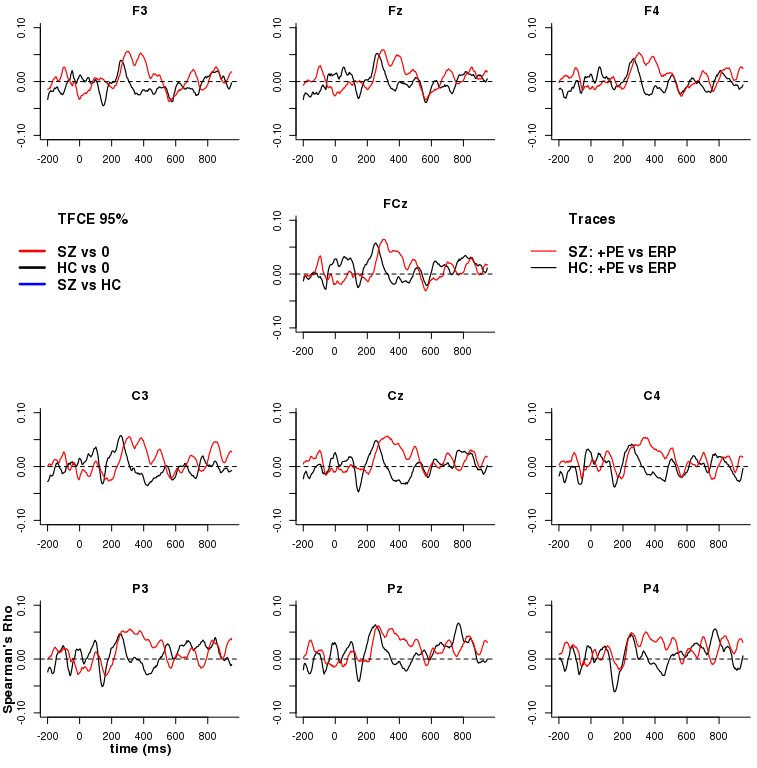

Supplement: S3 Fig — Spearman's correlation between voltage and +ve PE. Correlations between +ve PE and voltage were calculated for each person across all trials at each time point then group averaged. (TIF) [file pone.0152781.s003.tif]

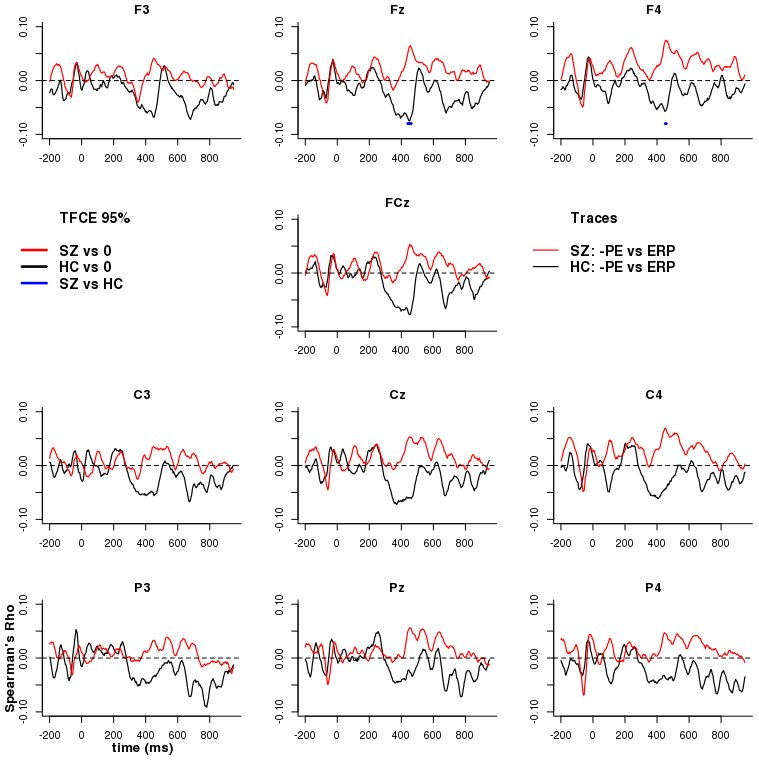

Supplement: S4 Fig — Spearman's correlation between voltage and -ve PE. Correlations between PE and voltage were calculated for each person across all trials at each time point then group averaged. (TIF) [file pone.0152781.s004.tif]

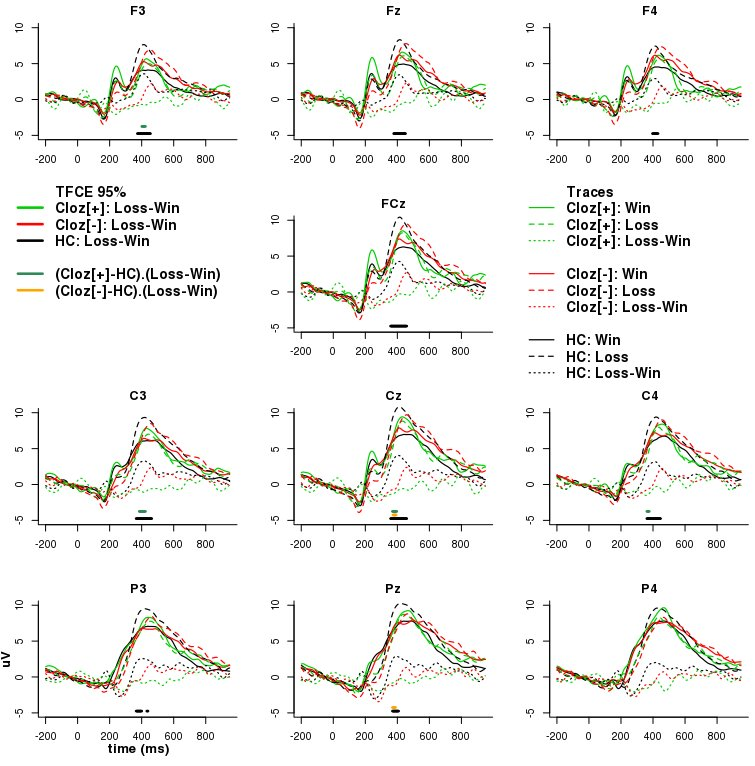

Supplement: S5 Fig — ERP to wins (thumbs up; solid lines), losses (thumbs down; dashed lines) and their contrast (dotted lines) for controls and patients by clozapine status. TFCE significance indicated by the solid horizontal bars at the bottom of each trace. (TIF) [file pone.0152781.s005.tif]

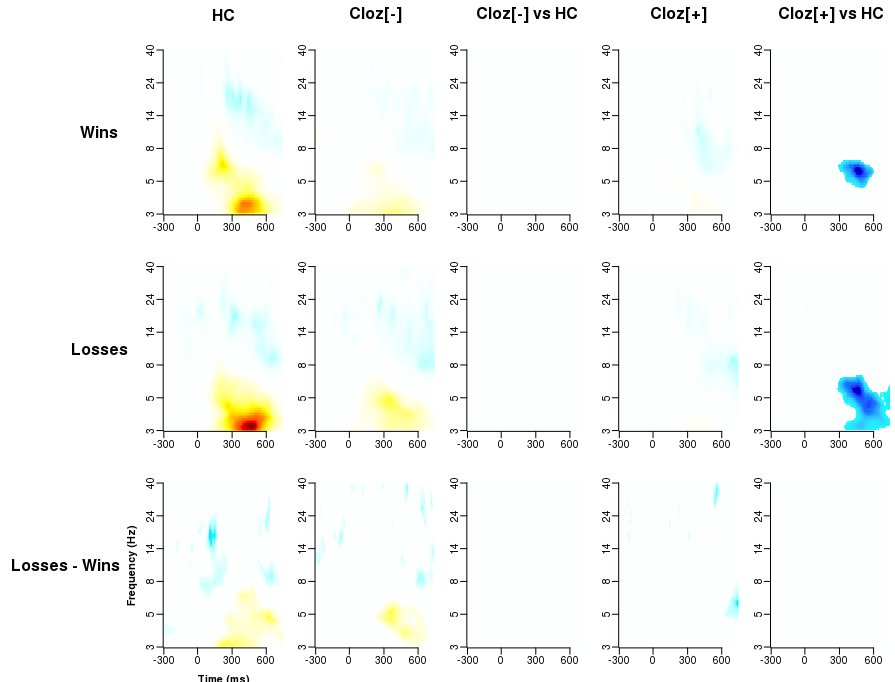

Supplement: S6 Fig — TFCE filtered time-frequency maps for wins, losses and their contrasts on the average of the three central midline electrodes Fz, FCz, and Cz. Colours are arbitrary, but symmetrical, mappings derived from the TFCE analysis scaled for best contrast. (TIF) [file pone.0152781.s006.tif]
