# Supplementary material for: Reduction of Pavlovian Bias in Schizophrenia: Enhanced Effects in Clozapine-Administered Patients
Source: PLoS One. 2016 Apr 4;11(4):e0152781. doi: 10.1371/journal.pone.0152781 (PMC4833478; doi:10.1371/journal.pone.0152781)
Supplement: S1 File — Includes information on RL modelling, TFCE, neuropsychological assessment methods and Bayesian analysis details. (DOC) [file pone.0152781.s007.doc]

Supplementary material for Albrecht et al.:

**Reduction of Pavlovian bias in schizophrenia: Enhanced effects in clozapine-administered patients**

**Modelling**

Models were adapted from previous modelling experiments using the Pavlovian bias task [1,2]. The models were built iteratively with increasing complexity (designated as M1 – M6). At each iteration, model fit was evaluated to determine whether increasing complexity improved the fit and captured important features of the data, model fits are presented in Table 2 of the main text. Reinforcements, were entered into the model as:

(1)

for punishment, neutral and reinforcement feedback respectively. The simplest model M1 possessed two free parameters: a single sensitivity for reward and punishment ***ρ*** and a learning rate ***ε***. These were used to update the state action value ***Q*** according to a delta rule, for action (Go, Nogo) and stimulus valence (Win, Avoid) state for trial *t*  (i.e., Go-to-Win, Go-to-Avoid, Nogo-to-Win, Nogo-to-Avoid):

(2)

A softmax function converted state action values ***Q*** for action options Go and NoGo into a single probability of action for each stimulus:

(3)

M2 included a third parameter *ξ*, that modulated the action probability of the state (P[a_t_|s_t_]) to allow for irreducible noise:

(4)

M3 included a fourth parameter *b*, to reflect the innate action preference of a Go response regardless of valence:

(5)

M4 allowed for differential sensitivities for rewards and punishments, *ρ^rew^* and *ρ^pun^*:

(6)

M5 included a fifth parameter *π*, reflecting the Pavlovian bias which modifies state action values according to their overall history of reinforcement or punishment across actions, *V_t_[S_t_]*, which is then added to the Go state action value, *Q_t_[Go|s_t_],* along with the go bias, *b,* according to:

(7)

M6 extended M5 by having separate group level estimates over all parameters for controls and patients.

Previous applications of this model used hierarchical expectation maximisation to estimate the free parameters for each participant. We used hierarchical Bayesian estimation, which tended to outperform slightly the expectation maximisation method on simulated parameter recovery tests. Hierarchical models in general have the advantage of shrinking individual level estimates towards a common value, creating a good compromise between highly variable individual level estimates and pooling all participants within a group to the one estimate. Hence, hierarchical models use all the available data for each parameter estimate.

The Priors used were mildly informative and were derived from the estimates from Table 1 in Cavanagh et al. [1] except with a modestly increased variance, to allow for differences between samples, e.g., age differences and diagnosis. The same priors were used for both patients and controls, so as to not bias towards an effect of diagnosis. Priors at the group level *x* (where *x = SZ, HC)* were specified as follows:

ρ^rew^_x_ ~ N(2,3)

ρ^pun^_x_ ~ N(2,3)

ε_x_ ~ N(0,3)

ξ_x_ ~ N(3.5, 2)

*b*_x_ ~ N(0,3)

π_x_ ~ N(0,3)

where, ρ^rew^_x_ = reward sensitivity, ρ^pun^_x_ = punishment sensitivity, ε_x_ = learning rate, ξ_x_ = irreducible noise, *b*_x_ = go bias, and π_x_ = Pavlovian bias.

These group level priors informed the participant level priors, such that for participant *y* in group *x*:

ρ^rew^_x,y_ ~ exp(*N*(ρ^rew^_x_, SD^rew^_x_)); SD^rew^_x_ ~ Cauchy(0,2)

ρ^pun^_x,y_ ~ exp(*N*(ρ^pun^_x_, SD^pun^_x_)); SD^pun^_x_ ~ Cauchy(0,2)

ε_x,y_ ~ 1/(1 + exp(- *N*(ε_x_, SD^ε^_x_))); SD^ε^_x_ ~ Cauchy(0,2)

ξ_x,y_ ~ 1/(1 + exp(- *N*(ξ_x_, SD^ξ^_x_))); SD^ξ^_x_ ~ Cauchy(0,2)

*b*_x,y_ ~ *N*(*b*_x_, SD*^b^*_x_); SD*^b^*_x_ ~ Cauchy(0,2)

π_x,y_ ~ *N*(π_x_, SD^π^_x_); SD^π^_x_ ~ Cauchy(0,2)

The Hamiltonian Monte Carlo sampler Stan [3] within R [4] was used to fit the models. The widely applicable information criteria (WAIC) was used to estimate improvements of fit for models of increasing complexity [5,6]. This was supplemented by the Bayesian Information Criteria (BIC). Convergence was monitored with the Gelman-Rubin statistic [7] (all participant's parameters < 1.1, more than 95% less than 1.01) and the number of effective samples was relatively high for each participant (more than 90% participants > 1000 and 95% > 700). Two participants were fitted with a low number of effective samples, one was characterised by a bimodal posterior in some of the parameters while there were no obvious pathologies in plots of the posterior over samples for the second participant. Each individual's parameter estimates were extracted using the median of the posterior distribution for further analysis.

**Symptom and Neuropsychological Assessment**

Participants were administered the Wechsler Abbreviated Scale of Intelligence (WASI; (8)), the Wechsler Test of Adult Reading (WTAR; [9]) and the MATRICS Consensus Cognitive Battery (MCCB; [10]). Participants with schizophrenia were further administered the Scale for the Assessment of Negative Symptoms (SANS; [11]), and the Brief Psychiatric Rating Scale (BPRS; [12]).

**Threshold Free Cluster Enhancement**

The TFCE value is an integral of all cluster cluster extents by cluster-heights and calculated across dimensions *x* and *y* according to:

$TFCE_{x,y}=\int_{h=0}^{h=max\left(h\right)} e_{x,y}^{E}*h_{x,y}^{H}.dh$ (8)

where *e* represents the number of neighbours above threshold and *h* represents the threshold. The weighting factors for extent and height, *E* and *H*, were set at 0.5 and 2 respectively. In practice, the integral is approximated in steps, with step size *dh* for analyses using t-statistics set at 0.1 and for correlation statistics set at 0.02. Positive and negative t-statistics or correlation coefficients were split into positive and negative matrices.

Violations of test assumptions and type I error rates were addressed using permutation statistics. For within subject contrasts, signs were randomly permuted, for between subjects contrasts, diagnostic category was randomly permuted. From 1000 permutations for each analysis, the maximum t-statistic from each permutation was stored forming an empirical null distribution. The null distribution was then used to filter the TFCE map obtained from the original non permuted data set at an alpha of 0.05, i.e., where the original TFCE scores were above 95% of the maximum permuted scores.

**Bayesian repeated measures ANOVA models and t-tests**

Bayesian split-plot ANOVA like models were conducted in R using the additional packages 'rjags' to link with the Gibbs sampler 'JAGS'. The analysis scripts were adapted from the split-plot scripts by Kruschke [15] to include heterogeneity of variance by fitting separate SD parameters for each group and stimulus type. A t-distribution was also included in the model for the error distribution with the degrees of freedom parameter set at 4, because it provides more robust estimates and handles skewed data better compared to the use of a normal distribution [16]. Hierarchical priors were used for each of the parameters of interest: the main effect of diagnosis, the main effect of stimulus type, the interaction between stimulus type and group. The participant level ‘random’ effects were described by a series of normal distributions centred on 0 with a standard deviation (SD) described by a half-Cauchy distribution [17]. The scale parameter for all half-Cauchy SD priors was estimated from the data with the prior on the scale parameter described as a uniform distribution between 0 and 1,000. The prior on the normality parameter (degrees of freedom parameter for the *t*-distribution) for the durations model was described by an exponential distribution centred on 30. All priors used for the Bayesian ANOVAs could be described as mildly informative on the scale of the data. For more information on the priors see [15].

For the split-plot models, a total of 1,000 adaptation steps were used to tune the samplers, 2,000 burn-in steps were discarded before taking 50,000 samples from the posterior spread across four chains. All chains showed good convergence of the final parameters and a high number of effective samples.

Similarly, two-sample t-tests were analysed using Bayesian t-tests from the 'BEST' package for R [18]. Briefly, the *t*-test from this package uses a robust version again via inclusion of a *t-*distribution to model the error distribution. In addition, the standard deviations are estimated for each group, thereby allowing a robust two sample contrast with unequal variances. The default priors and model structure from the 'BEST' package were used for this method.

95% highest density intervals of the posterior (95% HDI) were extracted from all models for statistical inference.

**References**

1. Cavanagh JF, Eisenberg I, Guitart-Masip M, Huys Q, Frank MJ. Frontal Theta Overrides Pavlovian Learning Biases. J Neurosci. 2013 Aug 5;33(19):8541–8.

2. Guitart-Masip M, Huys QJM, Fuentemilla L, Dayan P, Duzel E, Dolan RJ. Go and no-go learning in reward and punishment: Interactions between affect and effect. NeuroImage. 2012 Aug 1;62(1):154–66.

3. Stan Development Team. Stan: A C++ Library for Probability and Sampling [Internet]. 2013 [cited 2013 Dec 11]. Available from: http://mc-stan.org/

4. R Development Core Team. R: A language and environment for statistical computing [Internet]. Vienna, Austria: R Foundation for Statistical Computing; 2013. Available from: http://www.R-project.org

5. Watanabe S. A Widely Applicable Bayesian Information Criterion. J Mach Learn Res. 2013 Mar;14(1):867–97.

6. Gelman A, Hwang J, Vehtari A. Understanding predictive information criteria for Bayesian models. Stat Comput. 2014;24(6):997–1016.

7. Gelman A, Rubin DB. Inference from Iterative Simulation Using Multiple Sequences. Stat Sci. 1992 Nov 1;7(4):457–72.

8. Wechsler D. Wechsler abbreviated scale of intelligence. Psychological Corporation; 1999.

9. Wechsler D. Wechsler Test of Adult Reading: WTAR. San Antonio, TX: The Psychological Corporation; 2001.

10. Nuechterlein KH, Green MF, Kern RS, Baade LE, Barch DM, Cohen JD, et al. The MATRICS Consensus Cognitive Battery, Part 1: Test Selection, Reliability, and Validity. Am J Psychiatry. 2008 Feb 1;165(2):203–13.

11. Andreasen NC. The Scale for the Assessment of Negative Symptoms (SANS). Iowa City, IA: The University of Iowa; 1984.

12. Overall JE, Gorham DR. The Brief Psychiatric Rating Scale. Psychol Rep. 1962;10:799–812.

13. Mensen A, Khatami R. Advanced EEG analysis using threshold-free cluster-enhancement and non-parametric statistics. NeuroImage. 2013 Feb 15;67:111–8.

14. Pernet CR, Latinus M, Nichols TE, Rousselet GA. Cluster-based computational methods for mass univariate analyses of event-related brain potentials/fields: A simulation study. J Neurosci Methods. 2015;250:85–93.

15. Kruschke JK. Doing Bayesian Data Analysis: A tutorial with R and BUGS [Internet]. Burlington USA: Academic Press Elsevier; 2011 [cited 2012 Jul 6]. Available from: http://cognitivesciencesociety.org/uploads/2011-t2.pdf

16. Kruschke JK. Bayesian Estimation Supersedes the t Test. J Exp Psychol Gen. 2013;142:573–603.

17. Gelman A, Hill J. Data analysis using regression and multilevel/hierarchical models [Internet]. Cambridge University Press New York; 2007 [cited 2012 Jul 6]. Available from: http://www.imamu.edu.sa/Scientific_selections/abstracts/Math/Data%20Analysis%20Using%20Regression%20and%20MultilevelHierarchical%20Models.pdf

18. Kruschke JK, Meredith M. BEST: Bayesian Estimation Supersedes the t-Test. 2014.
